# Supplementary material for: Identification of transporter-dependent capsular loci associated with the invasive potential of Escherichia coli
Source: Nat Microbiol. 2026 Mar 25;11(5):1205–16. doi: 10.1038/s41564-026-02283-w (PMC13171598; doi:10.1038/s41564-026-02283-w)
Supplement: Supplementary file 1 — Reporting Summary [file 41564_2026_2283_MOESM1_ESM.pdf]

Corresponding author(s): Rebecca A. Gladstone
Jukka Corander

Last updated by author(s): 2025/12/31

Reporting Summary

Nature Portfolio wishes to improve the reproducibility of the work that we publish. This form provides structure for consistency and transparency in reporting. For further information on Nature Portfolio policies, see our Editorial Policies and the Editorial Policy Checklist.

Statistics

For all statistical analyses, confirm that the following items are present in the figure legend, table legend, main text, or Methods section.

n/a	Confirmed	
☐	☒	The exact sample size (*n*) for each experimental group/condition, given as a discrete number and unit of measurement
☐	☒	A statement on whether measurements were taken from distinct samples or whether the same sample was measured repeatedly
☐	☒	The statistical test(s) used AND whether they are one- or two-sided<br>*Only common tests should be described solely by name; describe more complex techniques in the Methods section.*
☐	☒	A description of all covariates tested
☐	☒	A description of any assumptions or corrections, such as tests of normality and adjustment for multiple comparisons
☐	☒	A full description of the statistical parameters including central tendency (e.g. means) or other basic estimates (e.g. regression coefficient) AND variation (e.g. standard deviation) or associated estimates of uncertainty (e.g. confidence intervals)
☐	☒	For null hypothesis testing, the test statistic (e.g. *F*, *t*, *r*) with confidence intervals, effect sizes, degrees of freedom and *P* value noted<br>*Give P values as exact values whenever suitable.*
☒	☐	For Bayesian analysis, information on the choice of priors and Markov chain Monte Carlo settings
☐	☒	For hierarchical and complex designs, identification of the appropriate level for tests and full reporting of outcomes
☐	☒	Estimates of effect sizes (e.g. Cohen's *d*, Pearson's *r*), indicating how they were calculated

Our web collection on statistics for biologists contains articles on many of the points above.

Software and code

Policy information about availability of computer code

Data collection: We collected data using a COBS indexed database of 661k bacterial genomes (Blackwell et al 2021, https://ftp.ebi.ac.uk/pub/databases/ENA2018-bacteria-661k/ https://github.com/graceblackwell/661K_query_indexes accessed July 2023).

Data analysis:
In Silico PCR https://github.com/simonrharris/in_silico_pcr
Bakta https://github.com/oschwengers/bakta/releases/tag/v1.11.4
Panaroo https://github.com/gtonkinhill/panaroo/releases/tag/v1.5.2
ISEscan https://github.com/xiezhq/ISEScan/releases/tag/v1.7.2.3
PopPUNK https://github.com/bacpop/PopPUNK/releases/tag/v2.6.5
Kaptive3 https://github.com/klebgenomics/Kaptive v3.0.0b5
Custom R v4.4.1 code: https://github.com/rgladstone/EC-K-typing/releases/tag/v3.0.0
Phandango https://github.com/jameshadfield/phandango/releases/tag/v0.5.0
Microreact https://doi.org/10.1099/mgen.0.000093
SRST2 https://github.com/katholt/srst2/releases/tag/v0.2.0
Gubbins https://github.com/nickjcroucher/gubbins/releases/tag/v3.4.3
pairSNP https://github.com/gtonkinhill/pairsnp/releases/tag/v0.0.1
BactDating https://github.com/xavierdidelot/BactDating/releases/tag/v1.1
SKA https://github.com/bacpop/ska.rust/releases/tag/v0.4.1
Mash https://github.com/marbl/Mash/releases/tag/v2.3
Shovill https://github.com/tseemann/shovill/releases/tag/v1.1.0
MSweep https://github.com/PROBIC/mSWEEP/releases/tag/v2.2.0
Thermisto https://github.com/algbio/themisto/releases/tag/3.0.0

Prokka https://github.com/tseemann/prokka/releases/tag/v1.14.5
Demix https://github.com/tmaklin/coreutils_demix_check/releases/tag/v0.3.2

For manuscripts utilizing custom algorithms or software that are central to the research but not yet described in published literature, software must be made available to editors and reviewers. We strongly encourage code deposition in a community repository (e.g. GitHub). See the Nature Portfolio guidelines for submitting code & software for further information.

Data

Policy information about availability of data

All manuscripts must include a data availability statement. This statement should provide the following information, where applicable:

- Accession codes, unique identifiers, or web links for publicly available datasets
- A description of any restrictions on data availability
- For clinical datasets or third party data, please ensure that the statement adheres to our policy

This study used published data. Accession codes are provided for all isolates analysed in this manuscript in the Supplementary Data. The babybiome assemblies are deposited at https://zenodo.org/records/14000489).

Research involving human participants, their data, or biological material

Policy information about studies with human participants or human data. See also policy information about sex, gender (identity/presentation), and sexual orientation and race, ethnicity and racism.

Reporting on sex and gender	Sex was collected for the BSAC Bacteraemia Resistance Surveillance Programme (https://bsac.org.uk/resistance-surveillance/) from hospital records and paired with the genomic data. Sex was considered in when looking at the distribution of K-types across age groups.
Reporting on race, ethnicity, or other socially relevant groupings	No information on race, ethnicity or other socially relevant groupings were available.
Population characteristics	Isolates were collected by BSAC from all age groups.
Recruitment	The BSAC collection consisted of isolates submitted to a Bacteraemia Resistance Surveillance Programme (https://bsac.org.uk/resistance-surveillance/) between 2001–2017 by 11 hospitals across England. From each hospital, the first 10 isolates (when available) for each year were included into the study.
Ethics oversight	The BSAC collection is now housed at the University of Dundee. Advice was sought from the Senior Clinical Research Governance Manager at the Health and Clinical Services, University of Dundee who supported the use of the age and sex data.

Note that full information on the approval of the study protocol must also be provided in the manuscript.

Field-specific reporting

Please select the one below that is the best fit for your research. If you are not sure, read the appropriate sections before making your selection.

☒ Life sciences ☐ Behavioural & social sciences ☐ Ecological, evolutionary & environmental sciences

For a reference copy of the document with all sections, see nature.com/documents/nr-reporting-summary-flat.pdf

Life sciences study design

All studies must disclose on these points even when the disclosure is negative.

Sample size	Phenotyping: n=150/3254 isolates representing different K-loci were phenotyped.<br>Invasiveness: BSI n=1840 carriage n=852
Data exclusions	For the invasiveness analysis: Data from disease in 2001-2002 was excluded due to large expansions in ST131 and ST69 in this time frame. Carriage data was filtered to one representative of a K-lineage within a family group to ensure independence. Data was filtered only to include K-loci found in >20 isolates, with >5 isolates of the infection and carriage groups each.
Replication	Phenotype concordance is reported in the supplementary Data, K-phenotyping has fallen out of use because it is laborious and subjective. Different subsamples of the invasiveness data gave the same overall findings.
Randomization	Invasiveness analysis used the isolation source as the experimental groups. Age and sex could not be included as a covariate so this is discussed as a limitation.
Blinding	There was no blinding. The study is observational. Researchers collected samples that inherently belonged to one group or the other based on the source patient's status. The "group allocation" was a fixed characteristic of the sample.

Reporting for specific materials, systems and methods

We require information from authors about some types of materials, experimental systems and methods used in many studies. Here, indicate whether each material, system or method listed is relevant to your study. If you are not sure if a list item applies to your research, read the appropriate section before selecting a response.

Materials & experimental systems

n/a	Involved in the study
☒	☐ Antibodies
☒	☐ Eukaryotic cell lines
☒	☐ Palaeontology and archaeology
☒	☐ Animals and other organisms
☒	☐ Clinical data
☒	☐ Dual use research of concern
☒	☐ Plants

Methods

n/a	Involved in the study
☒	☐ ChIP-seq
☒	☐ Flow cytometry
☒	☐ MRI-based neuroimaging

Plants

Seed stocks	NA
Novel plant genotypes	NA
Authentication	NA
